# Adipose-derived stem cells: selecting for translational success

**DOI:** 10.2217/rme.14.72

**Published:** 2015

**Authors:** Kavan S Johal, Vivien C Lees, Adam J Reid

**Affiliations:** 1 Blond McIndoe Laboratories, Institute of Inflammation & Repair, School of Medicine, University of Manchester, M13 9PT, UK; 2 University Hospital South Manchester, Southmoor Road, Manchester, M23 9LT, UK

**Keywords:** adipose, adipose-derived stem cells, clinical translation, cluster of differentiation markers, fat grafting, serum-free media, stromal vascular fraction, subpopulation selection

## Abstract

We have witnessed a rapid expansion of *in vitro* characterization and differentiation of adipose-derived stem cells, with increasing translation to both *in vivo* models and a breadth of clinical specialties. However, an appreciation of the truly heterogeneous nature of this unique stem cell group has identified a need to more accurately delineate subpopulations by any of a host of methods, to include functional properties or surface marker expression. Cells selected for improved proliferative, differentiative, angiogenic or ischemia-resistant properties are but a few attributes that could prove beneficial for targeted treatments or therapies. Optimizing cell culture conditions to permit re-introduction to patients is critical for clinical translation.

Significant scientific grounding in translational stem cell research combined with the more recent expansion of adipose tissue plasticity has identified adipose-derived stem cells (ASCs) as an important tool in regenerative medicine. However, building on lessons learned in other stem cell modalities, the need to characterize cell populations appears to be critical to enable selection of those groups that will maximize laboratory and potentially clinical outcomes. More accurate delineation of ASC subpopulations is also likely to be critical to patient safety where ASC augmentation is envisaged in a clinical context. A natural precedent for investigating ASC use in reconstructive surgery involving adipose tissue has already been set; however, it appears that ASCs may play an important role in translational research in a spectrum of other clinical specialties.

## Adipose tissue

At a cellular level, adipose tissue consists of mature adipocytes surrounded by fibroblasts, nerves, endothelial cells, immune cells and preadipocytic cells contained within a stromovascular cell network [1]. Enzymatic digestion of adipose tissue, specifically lipoaspirate, generates a heterogeneous population of adipocyte precursors within a pellet of cells termed the stromal vascular fraction (SVF). Increasing focus now falls on the capacity of such adipose-resident cells to undergo multilineage differentiation in a manner we have learned to recognize as typical of stem cells.

Origins of adipose tissue can be traced to the embryonic mesoderm, from where mesenchymal stem cells (MSCs) possess the ability to differentiate not only into adipocytes, but also chondrocytes, myocytes, osteoblasts [2] or indeed a host of other cell types by means of transdifferentiation. Dissection of the regulatory pathway from MSC to mature adipocyte is complex; the lipid-transcriptional factor PPARγ is likely to have a central role [3,4], with varying importance attached to (not exclusively) CCAAT/enhancer-binding proteins (C/EBPs) [5], lipoprotein lipase, Krüppel-like factors, early growth response 2 (Krox20), insulin and IGF-1 [6].

## Stem cells

Ongoing ethical dilemmas and concerns over oncogenesis in patients have restricted clinical use of embryonic stem cells [7]; however its more mature sibling, the multipotent adult (or somatic) stem cell, has far greater translational potential. In particular, hematopoietic stem cells (HSCs) from bone marrow have seen unparalleled clinical interest and therapeutic use [8,9], but HSCs remain inherently difficult to expand *in vitro* and even stringent isolation protocols for enriched HSCs can produce cells that do not demonstrate pluripotency or self-renewal. Within bone marrow, HSCs are surrounded by supporting bone marrow stromal cells (BMSCs) [10], a subpopulation of which possess mesenchymal tissue-regenerating properties [11]. Such naturally multipotent and self-renewing cells, defining MSCs, are not restricted to bone marrow alone and their presence in large quantities throughout connective tissues of the body is known to include synovia, tendons, skeletal muscle and adipose tissue alike [10].

Despite numerous studies attempting to define MSCs by site, surface markers or differentiation capacity, a standard accepted definition is lacking. The International Society for Cellular Therapy (ISCT) has previously defined three pre-requisites for consideration of a MSC: plastic adherence in standard culture conditions; expression of cell surface markers CD105, CD73, CD90 and lack of expression of CD45, CD34, CD14 or CD11b, CD79α, CD19, HLA-DR; and ability to differentiate into osteoblasts, adipocytes and chondroblasts *in vitro* [12]. Of particular note is the documented absence of CD34 on MSCs, a marker discussed later in the review due to its presence in SVF from adipose tissue and plastic-adherent ASCs. This is now recognized in the updated ISCT statement [13].

Relative ease of expansion by well-described protocols, ability for induced-differentiation into a host of cell lines *in vitro* and without the ethical concerns attributed to embryonic stem cells raise the attractiveness of the MSC for clinical applications; bone, liver, cardiac, skeletal muscle and CNS applications are at various stages of translation, including clinical trials [14].

### Adipose-derived stem cells

#### History, terminology & characterization

Investigation and clinical use of BMSC is now standard; however, concerns over acceptability of harvesting techniques and potentially low cell yields (1 in 10^5^ MSCs in culture adhere after initial plating [11,15]) have driven the search for alternative autologous MSC sources. The identification of multipotent precursor cells within processed lipoaspirates (PLAs) from human adipose tissue, building from well-established lessons in stem cell biology, has offered an alternative source [16]. Characterization of such populations has revealed remarkable phenotypic similarities to BMSCs [11], while being accessed by a considerably more tolerated harvesting procedure. More specifically, expression of CD markers classically associated with MSCs, together with the absence of numerous others including those of hematopoietic origin such as CD106, has added weight to the argument for a unique ASC population [17].

A catalog of studies attempting to characterize this heterogeneous cell population have followed (Table 1), with considerable similarities in SVF expression profiles noted by authors [18,19]. Once in culture, successful *in vitro* differentiation to adipogenic, chondrogenic, osteogenic and even neural end states has been described, confirming multilineage capacity and application of the term, ASCs. This is important to distinguish from the relatively heterogeneous population of SVF cells present after immediate tissue digestion by collagenase. Lack of uniformity in cellular characterization is likely a key contributing factor to the many and varied terminologies applied to these populations [20]: PLA cells, preadipocytes, SVF cells, adipose-derived adult stem cells, adipose stromal cells (also previously termed ASCs), adipose mesenchymal stem cells and adipose tissue-derived stromal cells among others. For the purposes of this review, the term ASC will be used to identify the specific subpopulation of cells described above only, as recommended by the consensus of the International Fat Applied Technology Society [21].

Temporal changes *in vitro* from SVF cells over multiple passages have also been noted [19,23]. The loss of hematopoietic markers CD11, CD14, CD45, CD86, HLA-DR with passage likely corresponds to a decreasing propensity for ASCs to be immunogenic with time in culture. Endothelial markers CD31 and CD144 are present at low levels in SVF but persist throughout. Perhaps most importantly, true mesenchymal markers (CD13, CD29, CD44, CD63, CD73, CD90 and CD166) all show progressively increased expression with many approaching 100% by passage 4 (P4). Other data have shown that even at P25, ASCs retain their multilineage differentiation capacity, although it is notably less than that at P0 [25].

Although some degree of conformity now exists in recognition of a baseline ASC phenotype within SVF, the presence and functional role of numerous other markers not classically associated with MSCs has become established as a rapidly expanding niche within the adipose stem cell arena. Subpopulations selected for improved proliferative, differentiative, angiogenic or ischemia-resistant properties are promising attributes that may prove to optimize targeted treatments or therapies.

### ASCs, MSCs & a perivascular origin

The precise location of stem cells within native tissue is of significant interest, not only for aiding processing and culture within the laboratory, but also for recognizing that progenitor cells from different sources exhibit similar lineage differentiation properties in the same environment. In fact, there is considerable evidence that MSCs may be derived from a common perivascular origin. Culture of perivascular cells from multiple tissues results in products expressing CD surface markers typical of MSCs (CD44, CD73, CD90 and CD105) that exhibit anticipated clonal proliferation and multilineage potential in suitable inductive conditions [26].

Specific examination by several authors appears to corroborate these data for ASCs. Immunofluorescence of adipose tissue sections identifies populations of CD34^+^ cells as being closely associated with vessels. Although small numbers of these are CD31^+^ and likely capillary endothelial cells, predominant perivascular CD34^+^/CD31^−^ populations are typical of ASCs and presumably pericytic. *In vitro* separation of CD34^+^/CD31^−^/CD144^−^ ASCs from endothelial cells (CD34^+^/CD31^+^/CD144^+^) by differential plastic adherence further supports distinct populations, with the majority of the ASC subset co-expressing MSC, pericytic and smooth muscle markers [27].

Other studies have employed immunohistochemistry techniques in combination with flow cytometry to further define population subsets (and their functional properties) based on direct relation to vessels. Pericytes, constituting the innermost layer of stromal cells contacting vessel endothelium, were described as CD31^−^/CD34^−^/CD45^−^/CD146^+^, while ‘supra-adventitial-adipose stromal cells’ localizing to the outer aspect of the vascular ring were characterized as CD31^−^/CD34^+^/CD45^−^/CD146^−^ cells. Both populations demonstrated adipogenesis, although the smaller-numbered pericytes more convincingly so [28].

Of note is the crossover between populations defined as pericytic or perivascular and those defined as ASC. As an example, a ‘supra-adventitial’ population phenotype [28] is identical to that defined as ASC in earlier work by others [29]. Many of the *in vivo* and *in vitro* studies discussed later in this review employ populations matching, either in part or completely, these population subsets.

### ASC location: macroscopic

Considering various anatomical depots, important differences have been noted in number and properties of harvested ASCs. Some authors have suggested the abdomen to have higher ASC yield than the hip/thigh, although found no difference in differentiation capacity between the two sites (for chondrogenic and osteogenic lineages) [30]. Others have compared gluteal and abdominal sourced ASCs, highlighting similar proliferation but identifying poor chondrocyte (both sites) and osteocyte/adipocyte (from abdominal ASCs) differentiation [31]. The expression of PPARγ, a known correlate of adipogenesis, has been tested on numerous anatomical sites and found to be highest in the arm, with the same study concluding ASCs from younger patients to be more proliferative and adipogenic. Here the authors also found superficial abdominal tissue to be the most resistant to apoptotic stimuli, which may have implications for survival *in vivo* [32].

Depth of adipose tissue harvest also appears to be critical to function, with ASCs derived from subcutaneous fat demonstrating more rapid growth and adipogenesis than those from visceral fat [33]. Comparing adipose in even closer proximity, namely superficial and deep abdominal fat, demonstrated that in male patients superficial depot ASCs differentiate faster (down an osteogenic line), although both layers were more efficient than in female patients highlighting potential intersex differences [34]. However, osteogenesis has been deemed to be more robust from the flank/thigh versus the arm/abdomen elsewhere, although this advantage was lost for flank depots when considering adipogenic differentiation [35]. The literature may provide conflicting arguments for anatomical depots, but as for standard liposuction harvesting techniques, the abdomen is likely to be popular as a preferred ASC harvest site owing to access, relative abundance and patient satisfaction.

### ASCs: subpopulation selection

Merits of selecting for surface markers expressed by ASCs are now well-recognized and a focus of investigation for several groups. Comparisons of sorting methods employed for subpopulation isolation are beyond the scope of this review, but largely encompass magnetic- or fluorescence-activated cell-sorting techniques.

### In vitro

Understanding overlap and significant differences in adipose-derived populations from bone marrow or hematopoietic lines has gathered interest. CD34, initially assumed hematopoietic-only marker, has seen a plethora of recent investigative studies (Table 2). Demonstration of presence of CD34 in the SVF has been confirmed by multiple authors [18,22,25,29]. CD34 detection, despite gradual decline with passage, is still possible after 10–20 weeks in culture [29].

Furthermore, improved proliferation in CD34^+^ versus CD34^−^ populations selected by flow cytometry has been demonstrated reproducibly [29,36]. The role of CD34 in adipogenesis appears less clear. CD34^−^ cells are described to contribute a more adipogenic and osteogenic cell lineage than positively selected cells [36]. This would seem to conflict with earlier work, suggesting CD34^+^/CD31^−^ represent a preferentially adipogenic subpopulation in stimulatory culture conditions and therefore likely contain key preadipocyte fractions. The absence of hematopoietic colony formation in stimulatory assays may refute the suggestion of CD34 as a hematopoietic-only marker that is present in digested adipose tissue only as a consequence of residual circulating blood; however, the presence of CD34 is widespread in many other tissues and this must also be considered [37].

The impact of co-culturing ASCs with mature adipocytes has been studied, to mimic the native environment within a transplanted fat graft and also the simulated ASC-augmented graft. Models employing subpopulations, specifically CD34, are described. Co-culture conditions resulted in significantly faster proliferation versus both standard and adipogenic medium for CD34^+^ selected cells [39].

CD34^+^/CD90^+^ populations are not constrained to adipogenic properties alone, but also show endothelial cell differentiation with high VEGF production from capillary-like structures [38]. Subpopulations selected for angiogenic properties may have potential benefits to autologous fat survival in reconstructive surgery, but also to a breadth of other clinical translations, including cardiac ischemia and vascular disease.

Establishing more detailed temporal alterations in expression may allow for optimal subpopulation selection. Indeed, data have suggested early expression of CD34 following initial ASC attachment in culture, followed by upregulation of CD105, CD146, CD271 and subsequent loss of CD34 expression [40]. Furthermore, considering these specific markers, subpopulations selected for the nerve growth factor receptor CD271 (p75NTR) have been shown to exhibit improved adipocyte, osteocyte and neuronal cell differentiation versus CD271^−^ cells [24]. Building on this work with CD271^+^ ASC, multilineage differentiation and improved proliferative rates have been observed when compared with unsorted plastic adherent cells and CD34^+^ populations [41].

The molecule CD105 (endoglin) is believed to have an intrinsic role in cellular attachment, aided by both CD146 and CD271 [40]. Positive selection of CD105 subpopulations for mesenchymal lineage differentiation has become established, most commonly for chondrogenic capacity. CD105^+^ cells show significantly greater chondrogenic potential *in vitro*, confirmed on tissue-culture plastic, gel-embedded sheets [42] and biodegradable scaffolds alike [43]. Scope for therapeutic use of such constructs for cartilage regeneration can be appreciated. Numerous other markers, including CD29 and CD73, are also implicated in improving chondrogenic pathway differentiation [45].

As the markers CD73, CD90 and CD105 are ISCT-defining of MSCs, it is logical they may characterize cells within an early undifferentiated state and thus offer greater potential for widespread tissue differentiation. To consider specific examples, CD73^+^ cells treated with a standard induction regimen (5-azacytidine) show significantly greater cardiomyocyte differentiation versus CD73- cells, as determined by myofibril and cardiac surface marker demonstration [46]. Mean while, CD105^+^ selection has been extended further afield to generate subpopulations capable of albumin production and ammonia detoxification, mimicking the role of primary human hepatocytes and offering hope to use of ASCs in regenerative hepatobiliary medicine [44].

Despite the widespread acceptance of characterizing MSC and subsequently ASC populations by CD markers, allowing some element of conformity among researchers, it is recognized these are unlikely to represent a definitive phenotype. Identification of embryonic and pluripotent stem cell markers within adipose-derived cell populations lends weight to the argument that a plethora of markers await exploration. SSEA-4, an early-expressed embryonic glycoprotein demonstrated in embryonic stem cells, marrow-derived MSCs and numerous oncological cell lines, is one such marker. High prevalence in human breast (and to a lesser extent abdominal) adipose tissue, with improved SSEA-4^+^ adipogenic and osteogenic differentiation versus mixed (unsorted) cell populations, has been shown. Interestingly, decreased expression in culture may indicate SSEA-4 as a relatively early, rather than late, MSC marker [47]. In appropriate media conditions, SSEA-4^+^ subpopulations have been demonstrated to progress along osteogenic and endothelial cell lineages, raising possibilities for tissue engineering translation, for example, vascularized bone constructs [48].

The *in vitro* characterization of ASC subpopulations offers insight into potential gain from selection; however, experience of translation to the clinical arena in medical research has warned us that changes observed on tissue-culture plastic may correlate poorly with those seen in the patient. Optimization of *in vivo* models is therefore critical to bridging the link from culture to clinic.

### In vivo

In comparison to the broader entity of stem cells, ASCs and particularly subpopulation selection remain in relative infancy with respect to quantity and quality of reproducible experimental data *in vivo*. Nevertheless, a steady influx of data pertaining to a breadth of tissue types is being seen.

### Adipose

Probably, the best understood subpopulation marker CD34 has the greatest volume of studies translating into animal models. Recently, the percentage of CD34^+^/CD146^−^/CD31^−^ cells present in SVF (defined as ‘ASC yield’) has been shown to directly correlate to fat graft volume retention in nude mice when small volumes are injected subcutaneously (Table 3) [49]. A relatively short study time of 8 weeks may not be sufficient for a direct clinical comparison as it is well recognized graft loss continues to occur after this point, but the usefulness of these data in predicting fat survival and alluding to potential autologous graft manipulation techniques should not be ignored.

Of the numerous studies discussed, many have utilized CD34 as a marker of proliferation and indeed plastic adherence. Further refinement within CD34 subpopulations by positive (rather than negative) co-selection of other markers may be of benefit in producing cells with targeted properties. Building on and confirming previous work demonstrating improved proliferation of CD34^+^/CD90^+^ subpopulations has led to successful seeding of sorted cells onto collagen sponge scaffolds *in vivo*. Increased adipogenic marker levels (PPARy and adiponectin) of CD34^+^/CD90^+^ seeded-constructs were confirmatory of observed macro- and micro-scopic adipocyte differentiation not observed with unsorted controls [51]. The true clinical relevance of results by the chosen 4-week end point may however be questioned again, necessitating longer study periods for substantiated conclusions.

Other studies have examined properties of subpopulations present in remarkably low quantities within SVF, including the use of CD24^+^ cells from within a murine Lin^−^CD29^+^CD34^+^Sca-1^+^CD24^+^ population that constituted only 0.08% of total SVF cells. Subsequent injection of GFP-labeled CD24^+^, CD24^−^ or unsorted cells into A-zip lipodystrophic mice demonstrated that only CD24^+^ cells were able to reconstitute adipose depots *in vivo* [52]. Later work analyzing CD24 content within transplanted cells has identified rapidly increasing percentages of CD24^−^ cells *in vivo*, from within a day after seeding. Given established deficits of CD24^−^ subpopulations forming adipose depots, it is likely that CD24^+^ adipocyte progenitors give rise to CD24^−^ cells, which may be further differentiated toward mature adipocytes [54]. Thus, selecting populations on the basis of high SVF prevalence, or *in vitro* proliferative and adipogenic capacity, may not in fact correlate to reciprocated *in vivo* results. Accurate mapping of temporal changes in expression following transplantation of a subpopulation may therefore be essential to dissecting the pathway from cell sorting to generated functional tissue.

Finally CD271, with robust multilineage capacity from *in vitro* data discussed above, has been utilized for adipocyte generation following subcutaneous injection of GFP-labeled ASCs loaded onto fibrin constructs [61]. Confirmation of blanket GFP expression in the newly formed adipose depots (not seen in controls) clearly attributes adipogenesis to donor rather than host cells.

### Bone

Translation *in vivo* has been observed for a host of other surface markers, including MSC markers that have shown *in vitro* promise. CD90^+^-enriched ASCs have recently been shown to undergo improved osteogenic differentiation over CD90^−^, CD105^+^ and unsorted cells. Results were corroborated by increased bone formation in CD90^+^ groups when seeded on hydroxyapatite-coated polylactic-*co*-glycolic acid scaffolds for the repair of calvarial defects in mice [55].

Despite these findings, the osteogenic role of CD105^+^ populations has been suggested elsewhere. Previously discussed *in vitro* data support CD105^+^ as a highly osteogenic subpopulation [43]. Other studies have further explored this subpopulation with flow cytometry separation of CD105 by high and low levels of expression (CD105^high+^ and CD105^low+^), demonstrating CD105^low+^ subpopulations constitute the more osteogenic population *in vitro* and *in vivo*. Significant differences were confirmed by serial micro-CT scanning of mice with parietal bone defects treated with CD105^low+^ cells on hydroxyapatite-coated polylactic-*co*-glycolic acid scaffolds, versus CD105^high^, unsorted and scaffold-only groups [56]. The latter study also concluded that co-selection of CD105^low+^/CD90^high+^ lends to a more osteogenic phenotype than CD105^low+^/CD90^low+^ *in vitro*; however, due to this population representing only 16% of ASCs, the authors excluded it from *in vivo* work on the premise that clinical translation was unlikely. This may support the role of CD90 in osteogenic differentiation as previously suggested [55]; however, it also opens a wider debate as to the importance of phenotypic drift once ASCs are cultured and the optimum time for sorting a specific subpopulation.

In the majority of studies discussed, pre-induction of ASCs in media specific for an end-target is routine prior to *in vivo* transplantation. However, cellular interaction in an animal model is inherently difficult to predict. Specific subpopulations within ASCs may be pre-defined to descend a particular lineage, which is only accelerated by the use of pre-induction protocols. Similarly, if the host microenvironment is the dictating factor in subpopulation behavior, then perhaps selection would occur regardless of intervention.

A recent study has compared osteogenesis among four different ASC populations: unsorted cells, cells sorted for CD44^+^/CD73^+^/CD90^+^/CD105^+^, unsorted cells subject to osteogenic differentiation media prior to experimental seeding (pre-induced unsorted) and pre-induced cells that had been further sorted for the above CD markers (pre-induced sorted). Of particular note, despite improved osteogenic capacity of pre-induced cells *in vitro*, after 8 weeks of *in vivo* seeding on β-tricalcium phosphate scaffolds, there were no significant differences in area of new bone formation relative to noninduced cells. Furthermore, cell sorting for any of the studied markers also failed to identify any differences (with or without pre-induction) [58]. On the basis of these findings, the authors advocate that neither cell sorting nor pre-induction is required, theoretically negating the need for extensive cell culture and sorting techniques. Results of this study should be noted; however, utilization of small patient numbers (n = 2 for number of donors) demands demonstration of reproducibility before conclusions can be drawn. The selection of cells at passage three is also somewhat unusual, with groups usually selecting directly from SVF or shortly after plating (Tables 2 & 3).

CD105 is not alone in being pursued as a translational osteogenic marker; several others including Stro-1 have been selected for assessment of *in vivo* bone regenerative capabilities. Regular detection in early characterization studies (Table 1) has converted to demonstration of osteogenic potential of Stro-1^+^ subpopulations. Degree of new bone formation in Stro-1^+^ sorted groups has been shown to be improved relative to certain markers (vs CD29, as confirmed by gene expression and micro-CT scanning of ASC-starch polycaprolatone constructs) [59] and at least equivalent to others (3G5, CD146 injected subcutaneously with hydroxyapatite/tricalcium phosphate powder into dorsum of mice) [60].

Furthermore, CD271 population subsets generating adipocytes above (Lin^−^CD271^+^Sca-1^+^) have also been employed for osteoblast formation within biphasic calcium phosphate-scaffolds *in vivo*.

### Muscle

Further demonstration of the effect of selecting more than one cell-surface marker can be seen with the pericyte marker neuron-glial 2 (NG2). Cells selected for CD34 and NG2 (CD34^+^/NG2^−^) have myogenic differentiation properties *in vivo* following seeding on cross-linked hyaluronic acid scaffolds in mice. However, CD34^+^/NG2^−^ cells lack all myogenic capabilities, but may reliably differentiate into adipocytes [53].

### Other

Subpopulations with a similar phenotype, notably CD34^+^/CD31^−^, have been employed previously for hypothesized vascular benefits. Intravenous tail vein injection of sorted cells into a mouse ischemic hind limb model resulted in time-dependent improved blood flow on laser Doppler versus CD34^−^ and no-cell controls, with immunohistochemistry confirming donor human cell incorporation into murine vessels [50].

Transdifferentiation potential of CD105^+^ subpopulations exhibited *in vitro* has been replicated *in vivo* for a number of uses, of which hepatocyte differentiation is of significant clinical interest. In specific induction cocktails, CD105^+^ ASCs undergo hepatocyte-like morphological changes with demonstration of associated functional properties. Furthermore, when transplanted in mice, incorporation into host (CCl4-damaged) livers with improvement of basic liver functions occurs [57].

Thus, it can be seen that a significant number of surface markers highlighted for *in vitro* subpopulation selection have undergone *in vivo* transition to date, although the breadth of data available (even within the same surface marker) creates difficulty in easily identifying the specific marker(s) most likely to have a desired end-function. However, the accelerated interest in implementation of ASCs, largely utilizing unsorted populations, has already become established across a number of clinical specialties.

## ASCs: bench to bedside

ASC populations employed clinically must be defined clearly by degree of cellular manipulation prior to patient re-introduction. Broadly, this encompasses unprocessed SVF cell populations (by definition not ASCs), unsorted and sorted ASCs. Debate over the correct nomenclature ascribed to cells has continued for some time, but it is the isolated, relatively homogenous, plastic-adherent multipotent precursor cells identified from human adipose tissue only that may correctly be termed ASC, as discussed above [21].

### ASCs in reconstructive surgery

Within the field of reconstructive surgery, the largest case series employing precursor cells from adipose tissue have been in the treatment of patients undergoing cosmetic breast procedures. In 2008, Yoshimura *et al.* expanded on their earlier work characterizing surface markers in lipoaspirate samples and adherent-ASCs [62] to release a 40-patient series of fat transfer procedures for breast augmentation, incorporating fat grafts supplemented with simultaneously extracted SVF cells in a process termed cell-assisted lipotransfer (CAL). Subjective clinician and patient satisfaction was reported, with centrifuged fat mixed with SVF cells (vs noncentrifuged and SVF injection groups) correlating to highest fat retention. The exact mechanism for these results is unexplained; of particular note is the use of SVF cells and not isolated ASCs with the authors postulating possible interactions between this heterogeneous population of cells and mature adipocytes [63].

Later work employing the CAL technique to address volume deficiency after implant removal in 15 patients who had developed capsular contracture reported a 40–80% graft take as determined by 3D photographic assessment. No complications (cyst formation, micro-calcification or oncological concerns) were detected on postoperative MRI or mammography [64]. The authors have extended their novel method to the treatment of facial lipoatrophy, a small six-patient series reporting subjective improvement (although results were not significant) in CAL versus non-CAL fat grafting techniques as determined by simple pre- and post-operative photographic assessment only [65].

Also falling within a reconstructive remit, ASCs have been employed for the treatment of end-stage radiation tissue necrosis in breast cancer patients, although the authors used non-PLA assumed to contain ASCs without SVF separation or culture [66].

In order to facilitate ‘bedside’ ASC-integration, groups have employed the use of specifically manufactured commercial products that utilize enzymatic digestion to deliver ‘adipose-derived regenerative cell-enriched grafts’. Negating the need for transport of tissue and *ex-vivo* culture is potentially appealing, although a true appreciation of absolute cell yield or composition is extremely difficult to ascertain. In a series of 67 patients who had undergone surgery to remove breast cancer but to preserve the unaffected breast tissue, the use of ‘ADRC-enriched’ fat grafts for reconstruction of the breast tissue defect resulted in high satisfaction levels reported by clinicians and patients alike [67]. However, the authors themselves recognized that objective volumetric analysis (MRI as an example) was lacking. Although it is acknowledged that statements on oncological safety cannot be determined currently (1-year follow-up at time of publishing), given the uncertainty relating to injected cellular composition and *in vivo* interaction, the question arises whether further basic scientific grounding is required before any future clinical introduction.

### ASCs & safety

One of the few studies examining effects of ASCs on breast cancer cells (*in vitro* and *in vivo* model) determined that ASCs do augment the growth of active, but not resting breast cancer cells. The authors stated that extrapolation of these results may suggest the potential for ASCs to promote breast tissue regeneration but should not affect the status of dormant residual cancer cells [68]. Other *in vivo* studies have identified increased proliferation of tumor cell lines, in both co-culture with ASCs and also when treated with ASC-conditioned media. Despite no increase in new tumor vessel formation, a decreased rate of apoptosis in ASC presence may suggest preference toward tumor growth in the ASC-supplemented environment [69].

In a separate murine model, co-engrafting of ASCs with active prostate cancer cells led to a greater than threefold increase in tumor volume in comparison to those grafted without ASC supplementation [70]. This may have therapeutic benefit in determining the role of ASCs in prostate and indeed many other cancers, but presently it is sufficient to continue to avoid use if any oncological concerns, current or previous, are expressed.

Very recently, human ASCs cultured with ‘triple negative’ breast cancer cell lines had no effect on growth in culture, but did stimulate metastases to other murine organs *in vivo* that were not seen in controls without ASCs. In one case, increased VEGF and microvessel density was observed, suggesting increased tissue angiogenesis that may be of concern in a tumor bed [71].

A concise review of studies assessing MSC effect (including human ASCs) on various tumor sizes, growth and metastases outlined the difficulties in ascertaining safety even at a preclinical stage. With a catalog of data suggesting that MSCs may promote or alternatively inhibit tumor growth, the authors correctly conclude that our current knowledge of mechanisms by which MSCs may exert their effects is still poorly understood, such that predictions on cell behavior and anticipated safety cannot be made. The authors do stress that there has been no evidence of tumor formation directly attributed to MSC use in all patients treated to date [72]. Clearly, further reproducible studies minimizing discrepancies in donor tissue, recipient cells, timing of MSC addition and monitoring parameters are required. Current data, however, are likely sufficient to preclude ASC-supplemented grafts in sites of previous oncological diagnoses until more is known about potential for locoregional or even metastatic recurrence.

### Cross-specialty ASC translation

Regardless, we have now seen the first landmark clinical trial of unsorted ASCs cultured *in vitro* prior to supplementation in subcutaneously injected fat grafts, albeit in healthy nononcological cohorts. A concentration of 20 × 10^6^/ml ASCs employed to enrich grafts resulted in significantly higher residual volumes at a 121-day end point (80.9 vs 16.3% in nonsupplemented control grafts), confirmed by serial MRI imaging and surgical removal of grafts at experimental completion [73]. The use of fat bolus injection versus conventional small-volume 3D layering of fat parcels (as described by Coleman [74]) and relatively small patient numbers (n = 10) is noted. However, the potential implications for reconstructive surgery procedures currently reliant on allogenic materials or free tissue transfer could be dramatic.

Aside from use as adjuncts to autologous fat grafting, expanded ASCs have also been employed in colorectal surgery for perianal fistula treatment since 2003 [75], with recent publication of a multicenter 24-patient study demonstrating 56% complete fistula closure with direct ASC infiltration in cases that had previously failed to respond to medical management [76]. Relying on improved osteogenesis with ASC supplementation, use has extended to include bone mineralization of titanium scaffolds in order to create a *de novo* vascularized bone flap for palatal reconstruction in craniofacial surgery [77].

Rapid expansion of interest in ASC translation is reflected in the breadth of specialties for which patients are currently being recruited. A recent search under the criteria ‘adipose stem cell’ reveals 109 clinical trials currently registered with the US Institute of Health [78], a marked increase from the 40 clinical trials available when accessing similar data in 2011 [79]. Musculoskeletal (osteoarthritis, rheumatoid arthritis), metabolic (Type 1 and 2 diabetes), neurological (multiple sclerosis, Parkinson’s disease, schizophrenia, ischemic stroke spinal cord injury), cardiac (myocardial infarction/congestive heart failure), respiratory (chronic obstructive pulmonary disease, acute respiratory distress syndrome) and hepatobiliary (liver cirrhosis) are but a few of the widespread applications for which ASCs are currently being sought. Of particular note is the intended use of unsorted cell populations for registered trials. However, with such active interest in subpopulation assessment following sorting *in vitro* and now *in vivo*, the final bridging step to ASC selection for use in the clinical arena now appears to be on the horizon.

Although clinical ASC subpopulation data are scarce, other sources of adult stem cells, particularly hematopoietic populations, have seen tremendous influx of subpopulations employed clinically. CD34^+^ content within stem cell transplants is widely used as a predictor of graft success and large doses of CD34^+^ purified peripheral blood cells have been administered to HLA-mismatched patients on the premise of selection-induced T-cell depletion with subsequently reduced risk of graft-versus-host disease [80]. The literature regarding surface marker selection in this field is extensive; however, the use of CD34 is far from limited to a hematopoietic therapeutic niche.

Significant CD34^+^ data may also be found in cardiac research, with Phase II trials of CD34^+^ intramyocardial infusion into infarcted tissue already completed. Reduction in angina frequency and improvement in exercise tolerance have been reported [81], with Phase III trials now registered for the treatment of refractory angina [82]. Purified CD34^+^ populations have also been directly compared via transendocardial versus intracoronary infusion techniques in the treatment of dilated cardiomyopathy, with the former correlating to improved left ventricular function and exercise capacity [83]. Mirroring the widespread system distribution of *in vivo* data, leukapheresis samples from patients with liver insufficiency have been isolated for CD34 using commercially available magnetic sorting techniques and subsequently infused under image guidance directly into the portal vein or hepatic entry [84]. The safe use of antibodies for such sorting techniques is critical in the clinical arena and although not discussed in detail here, can be found comprehensively reviewed elsewhere [85].

Should adipose tissue-based research progress in the manner observed for hematopoietic work, we are likely to see an expansion in ASC subpopulation trials pending clinical introduction. For this to be a measured reality, certain aspects of processing, culture (where applicable) and patient re-introduction of ASCs demand refinement.

### Refinements for clinical translation

Current use of ASCs, with or without *ex vivo* expansion, involves numerous steps, which individually and cumulatively have direct potential for patient harm. Strict regulation in the transfer and use of tissue is required to minimize risk. Sterility is paramount at all times, but particularly when laboratory processing and culture is undertaken. The increased implementation of commercial products that allow bedside subpopulation sorting prior to immediate use ‘on-table’ is suggested to reduce these risks; however, in return the ability to characterize and expand populations is lost. Of course, the logistical and financial effects of reducing patient re-operation should not be understated, nor the potential impact on patient satisfaction, all of which must be counterbalanced by significant start-up and training costs.

Where adipose tissue is removed from a clinical setting, assuming sterile handling and appropriate transportion methods, it is the cellular digestion and culture of tissue that introduces the greatest variables to the regenerative loop that ultimately returns to the patient. Protocols for ASC harvesting are well described and almost universally adopted. Of particular note is the use of xenogenic animal-derived products that introduce the risk of contamination and prompt serious biosafety considerations. Transfer of bacterial or viral infections [86], prions [87], allergy and anaphylaxis [88,89] are all of concern when using bovine derivatives such as fetal bovine serum (FBS) routinely employed in cell culture. Furthermore, initial FBS extraction techniques carry not only scientific, but potentially ethical and religious implications. A number of other products, to include collagenase and trypsin, may also be sourced from animal derivatives. The need to establish alternatives to xenogenic products, to allow for start-to-finish xeno-free protocols, has become a pressing reality in translational stem cell medicine.

### Adipose tissue digestion

The use of collagenase for adipose tissue digestion prior to SVF isolation is widely adopted. However, a number of current collagenase products available are not clearly defined to be devoid of animal products, therefore by definition may be xenogenic. Animal-free collagenase products are available, but their current use tends to be limited and may be prohibited by cost. When establishing alternatives or substitutes to any given product, unchanged cellular morphology, phenotype (to include surface marker characterization) and behavior in culture must be demonstrated. A recent study comparing research-grade collagenase (potentially containing animal products) with ‘animal origin-free’ and xeno-free lyophilisates established no differences in cell yield, proliferation, CD marker phenotype or differentiative capacity [90].

### Alternatives to FBS for ASC culture

A further challenge in *ex vivo* expansion of ASCs with clinical intent remains the identification of xeno-free alternatives to standard FBS-based media, devoid of bovine products and with reduced lot-to-lot variability. Numerous options have been considered for the culture of bone-marrow-derived MSCs; however, data concerning ASCs have been more limited. As the regenerative potential of ASCs now becomes more widely recognized, a parallel surge in clinical translatability studies is being seen.

Significantly improved ASC proliferation with retention of multilineage capacity has been demonstrated in commercially available serum-free xeno-free media [91,92], although of note is the requirement to pre-coat tissue culture flasks with relevant attachment substrates. Further work has identified similar results with xeno-free media comprised of knockout-DMEM supplemented with human serum albumin and other factors generated from synthetic, recombinant or human sources [93].

Most recently, authors have recognized the need for complete xeno-free protocols, with the use of human serum, human serum albumin and Tryple as alternatives to FBS and trypsin in the culture of ASCs [94]. Improved proliferation and maintenance of ASC phenotype without modification of gene expression was documented.

A host of other serum alternatives have been suggested for unique benefits, reduced immunogenicity using umbilical-cord-derived extracts as an example [95]. Proliferation was demonstrated to be comparable to standard FBS conditions. Thrombin-activated platelet-rich plasma, previously considered with bone-marrow MSC populations, has also been demonstrated alongside pooled human AB serum (AB-HS) for significantly higher (approaching threefold) ASC proliferative rates versus FBS [96]. Furthermore, markedly reduced population doubling time has been shown using DMEM supplemented with pooled human platelet lysates, again versus FBS controls [97]. None of the above studies identified significant change in surface marker expression in comparison to standard culture techniques, nor functional capacity as further assessed by differentiation.

Serum-free alternatives now command significant interest from the scientific and clinical community alike; however, the body of experimental data relating to ASCs still remains based on bovine serum. Each of the suggested products has merits and disadvantages, which are reviewed in extensive detail individually elsewhere. In general terms, the ideal culturing media should be cost effective, have minimal batch variability, low immunogenicity and be free of all animal, but also likely allogenic derivatives. Perhaps in time we will see the synthetic serum-free xeno-free product become the frontrunner in *ex vivo* ASC expansion.

## Conclusion & future perspective

Within the last decade, a great volume of laboratory data targeting unsorted ASCs to a spectrum of clinical problems has seen rapid escalation to clinical trials. However, we must not lose sight of the importance of adequate scientific grounding in both *in vitro* and *in vivo* models, before what may be potentially hasty progression to patient administration.

Within ASC research, the identification, characterization and selection of subpopulations have identified unique challenges with potentially even greater translational benefit. While the evidence for specific ASC selection as a therapy toward specific clinical scenarios is yet to be determined, this is likely to represent an important component of clinical advancement within this field over the next 5 years. Reconstructive surgery, cardiac, musculoskeletal and neurological medicine are a few of the areas that may profit.

Further well-conducted, reproducible data are required that will answer numerous questions, not least between immediate population sorting and re-introduction over *ex-vivo* expansion. For the latter, stringent adherence to good clinical practice and laboratory principles alike will be fundamental to ensure optimum patient outcomes.

## Figures and Tables

**Table 1:** Surface markers expressed in human and animal adipose lipoaspirates (degree of differentiation and number of passages is shown where relevant).

Positive markers	Negative markers	Source	Differentiation	Ref.
CD9, CD10, CD13, CD29, CD34, CD44, CD49e, CD54, CD55, CD59, CD105 and CD166, HLA-ABC	CD11a, CD11b, CD11c, CD14, CD18, CD31, CD45, CD50, CD56, CD62e, Stro-1, HLA-DR	Human SVF	*In vitro*: adipogenic, osteogenic	[18]

CD13, CD29, CD44, CD49d, CD71, CD90, CD105, SH2, SH3, Stro-1	CD14, CD16, CD31, CD34, CD45, CD56, CD61, CD62e, CD104, CD106	Human SVF	*In vitro:* adipogenic, osteogenic, chondrogenic, myogenic, neurogenic	[17]

CD29, CD44, CD90, CD105, c-Kit	CD14b, CD34, HLA-DR	Human ASC-P0 (72 h)	*In vitro*: adipogenic, osteogenic, chondrogenic	[22]

CD13, CD29, CD31, CD34, CD44, CD49a, CD63, CD73, CD90, CD144, CD146, CD166 (inc), VEGF, vWF	NA	Human SVF to P4	*In vitro:* adipogenic, osteogenic	[23]

CD11a, CD13, CD14 (dec), CD29, CD34, CD40 (inc), CD44, CD45(dec), CD54 (inc), CD63, CD73, CD80, CD86 (dec), CD90, CD166, CD31, CD144, ABCG2, HLA-ABC (inc), HLA-DR (dec)	NA	Human SVF to P4	NA	[19]

CD29, CD31, CD34, CD90, CD105, CD271, Sca-1	CD45 (very low)	Mouse SVF	*In vitro:* adipogenic, osteogenic, chondrogenic, myogenic, neurogenic	[24]

CD13, CD29, CD44, CD105, CD166	CD34, CD45, HLA-DR	Human P4, P20, P25	*In vitro*: adipogenic, osteogenic, chondrogenic, myogenic	[25]

ASC: Adipose-derived stem cell; NA: Not available; P: Passage; SVF: Stromal vascular fraction.

**Table 2:** Select subpopulation *in vitro* studies.

Surface marker	Surface marker subset	Source	Time of sorting	Sorting technique	Change in culture	Differentiation	Clinical translation	Comments	Ref.
CD34 and subsets	CD34^+^	Human	P0 (7 days)	FACS					[36]
	CD34^−^					Ad^+^, Os^+^	Fat grafting		
	CD34^+^/CD31^−^	Human	SVF	MACS		Ad^+^	Fat grafting	Preadipocyte population enriched in adipogenic culture	[37]
	CD34^+^	Human	SVF	FACS		Ad^+^Ch^+^Os^+^	Any		[29]
	CD34^+^/CD90^+^	Human	P0 (5–7 days)	FACS		Ad^+^	Fat grafting		[38]
	CD34^+^	Human	SVF	MACS			Any	Improved proliferation in adipocyte co-culture	[39]
	CD34^+^	Human	SVF	FACS	Dec		Any	Downregulation following ASC ‘activation’ during proliferation	[40]
	CD34^+^	Human	SVF	MACS		Ad^+^, Ch^+^, Os^+^	Any		[41]

CD271 and subsets	CD271^+^	Human	SVF	MACS		Ad^+^, Ch^+^, Os^+^		> than CD34	[41]
	CD271^+^	Albino mice	SVF	FACS		Ad^+^Os^+^Ne^+^	Any including nerve regeneration		[24]

CD105	CD105^+^	Human	SVF	FACS		Ad^+^Ch^+^Os^+^	Any		[29]
	CD105^+^	Mouse	SVF	MACS		Ch^+^	Cartilage repair		[42]
	CD105^+^CD105^−^	Human	SVF	MACS		Ch^+^, Os^+^Ad^+^	Any		[43]
	CD105^+^	Human	P3–5	MACS		Ad^+^, Ch^+^, Os^+^ Hepatic differentiation	Hepatobiliary regeneration		[44]
	CD105^−^	Human	SVF	MACS		Ad^+^	Fat grafting		[37]

Other select markers	CD31^+^/VEGF^+^/Flk-1^+^					EC^+^, high VEGF production	Vascular disease, cardiac ischemia		[38]
	CD105^+^/CD146^+^/CD271^+^	Human	SVF	FACS	Inc		Any	Upregulation following ASC ‘activation’ during proliferation	[40]
	Stro-1^+^	Human	SVF	MACS		Os^+^			[45]
	CD29^+^ CD105^+^					Ch^+^Ch^+^			
	CD49^+^, CD90^+^, C D271^+^					Os^+^, Ch^−^			
	CD73^+^					Ch^+^, Os^−^			
	CD73^+^	Mouse	P3	FACS		Cardiomyocyte differentiation	Cardiac ischemia		[46]
	SSEA-4	Human	SVF	MACS	Dec	Ad^+^Os^+^			[47]
	SSEA-4	Human	SVF	MACS		Os^+^En^+^	Vascularized bone constructs/tissue engineering	In osteogenic and endothelial conditions, respectively	[48]

Ad: Adipogenic; ASC: Adipose-derived stem cell; Ch: Chondrogenic; Dec: Decrease; EC: Endothelial cell; FACS: Fluorescence-activated cell sorting; Inc: Increase; M: Muscle; MACS: Magnetic-activated cell sorting; Ne: Neuronal; Os: Osteogenic; P: Passage; SVF: Stromal vascular fraction.

**Table 3:** Select subpopulation *in vivo* studies.

Surface marker	Population	Source	Model	Study time	Time of sorting	Sorting technique	Platform	Differentiation	Key results	Ref.
CD34 and subsets	CD34^+^	Human	Nude mice	8 weeks	NA	FACS	sc. injection		ASC yield predicts fat graft retention	[49]
	CD34^+^/CD31^−^	Human	Mouse	2 weeks	SVF	MACS	iv. ASC injection into ischemic hindlimb	EC^+^	Improved blood flow and neovascularization	[50]
	CD34^+^/CD90^+^	Human	Nude mice	4 weeks	P0	FACS	Collagen scaffold		Adipocyte differentiation in sorted ASCs on scaffolds	[51]
	Lin^−^CD29^+^CD34^+^ Sca-1^+^CD24^+^	Mouse	A-zip mice	12 weeks	SVF	FACS	Parametrial adipose tissue pad injection	Ad^+^	CD24^+^ cells reconstitute WAT adipose depots *in vivo*	[52]
	NG2^+^/CD34^+^ NG2^−^/CD34^+^	Human	Mouse	30 days	SVF	FACS	Cross-linked hyaluronic acid scaffold	M^+^ Ad^+^	NG2^+^ and NG2^−^ encode myogenic and adipogenic phenotypes, respectively	[53]

CD24	CD24^+^	Mouse	A-zip mice	6 weeks	SVF	FACS	sc. injection	Ad^+^	CD24^+^ adipocyte progenitors generate CD24^−^ adipocyte-committed cells *in vivo*	[54]

CD90	CD90^+^	Human	Mouse	8 weeks	P0 (36 h)	FACS	Calvarial defects in mice	Os^+^	CD90 > CD105 for *in vitro* osteogenic differentiation and calvarial defect repair *in vivo*	[55]

CD105	CD105^low+^ CD105^low+^/CD90^high+^ (*in vitro* only)	Human	Mouse	8 weeks	P0 (36 h)	FACS	Mice calvarial defects	Os^+^ Os^+^	CD105^low^ > CD105^high^ and US cells for osteogenic differentiation *in vivo*	[56]
	CD105^+^	Human	Mouse	24 h postinjection	SVF	MACS	Transplantation	Hepatic differentiation	CD105^+^ ASCs show host liver incorporation and improvement of liver function	[57]

Other select markers	CD44^+^, CD73^+^, CD90^+^, CD105^+^	Human	Mouse	8 weeks	P3	FACS	β-TCP scaffolds	Os^+^	No differences unsorted vs sorted osteogenesis (± osteoinduction)	[58]
	Stro-1^+^ CD29^+^	Human	Mouse	6 weeks	SVF	MACS	SPCL scaffold	Os^+^	Stro-1^+^ more osteogenic than CD29^+^ when pre-seeded on SPCL scaffolds *in vivo*	[59]
	Stro-1^+^ 3G5^+^CD146^+^	Human	Mouse	8 weeks	SVF	FACS	sc. injection with HA/TCP powder	Os^+^Ad^+^ (*in vitro* only)	Stro-1^+^/3G5^+^/CD146^+^ equally adipogenic and osteogenic, the latter confirmed *in vivo*	[60]
	Lin-CD271^+^Sca-1^+^	Mouse	Mouse	6 weeks	SVF	MACS	BCP scaffold in femoral defect	Os^+^	Sorted populations generate osteoblasts and mature adipocytes *in vivo*	[61]
							sc. injection of ASC–fibrin construct	Ad^+^		

Ad: Adipogenic; ASC: Adipose-derived stem cell; BCP: Biphasic calcium phosphate; EC: Endothelial cell; FACS: Fluorescence-activated cell sorting; HA: Hydroxyapatite; iv.: Intravenous; M: Muscle; MACS: Magnetic-activated cell sorting; Os: Osteogenic; P: Passage; sc.: Subcutaneous; SPCL: Starch polycaprolatone; TCP: Tricalcium phosphate; US: Unsorted; WAT: White adipose tissue.

## Executive summary

### Stem cells & adipose-derived stem cells

- Adipose-derived stem cells (ASCs) are a heterogeneous cell population, with properties that may be translated to clinical benefit including fat graft survival.
- ASC subpopulation selection for generating refined populations with targeted functions is likely advantageous.

### ASCs: bench to bedside

- A wealth of *in vitro*, *in vivo* and now early clinical data involving ASC subpopulation use have been seen within the last 3–5 years.

### Conclusion & future perspective

- Safe clinical translation demands rigorous laboratory grounding to ensure benefit without risk of harm, to include the optimization of all steps involved in *ex-vivo* selection and/or culture of human ASCs.
